# Carcinoma of the Uterus and Some of Its Problems

**Published:** 1911-10

**Authors:** W. F. Shallenberger

**Affiliations:** Atlanta, Ga.


### CARCINOMA OF THE UTERUS AND SOME OF ITS PROBLEMS.

W. F. Shallenberger, M. D., Atlanta, Ga.

It is not my purpose in this paper to go into a detailed discussion of carcinoma of the uterus. I wish to dwell especially upon the treatment of the inoperable cases and shall only touch upon the pathology and operative treatment. I have nothing new to offer, but I wish to call your attention particularly to the use of acetone in inoperable carcinoma of the uterus and to give my experience with it.

Carcinoma has been one of the few diseases that has held out against the advancement of Medical Science. But an enormous amount of work is being done on carcinoma to-day and it is being assailed on all sides and sooner or later the problem will be solved. Until then it is our duty as physicians to do all in our power to prevent and overcome this disease. We must be ever on the alert—ready to make an early diagnosis and not have to wait until the classical symptoms, haemorrhage and the foul discharge, are present. There is no one symptom peculiar to carcinoma alone, and we must keep this in mind and teach it to the women of this country. By educating the women to consult their physician upon the first manifestation of any abnormal pelvic symptom we shall be enabled to get the cases earlier. And it is important that we insist upon a thorough examination in all cases where any pelvic disturbance arises. Any irregularity of the cervix that bleeds on ordinary vaginal examination should be regarded as carcinoma or sarcoma until proved otherwise by a careful histological examination of a piece of the tissue. Any irregularity of the menstrual flow, especially an increase or intermenstrual bleeding, should be regarded with grave suspicion and at once investigated by an examination and a thorough curretage of the uterus for diagnostic purposes if necessary.

In Germany, Duerhssen and Winter have been doing much along the line of education for years, and in consequence their percentage of operative cures is much higher than in this country. Winter has been reaching the women through the press. Ethics and our so-called modesty make the question more difficult in this country.

It has even been suggested that all women present themselves for examination every three or four months, and this is not a bad idea.

There are certain prophylactic measures also which must be kept in mind. We know the important role that trauma plays in the occurrence of carcinoma. Trauma and repeated irritation. Thus we find carcinoma of the stomach developing in the scar of a healed ulcer many times, and carcinoma of the lip and breast following injury. So also lacerations of the cervix from childbirth with the resulting scars are a determining factor in carcinoma here. Only from 1% to 5% of carcinoma of the cervix occurs in nulliparous women. Early repair of all such lacerations or amputation of the cervix are the prophylactic measures to be adopted.

There are several varieties of carcinoma of the uterus. In the cervix the squamous-celled type is by far the most common and in itself constitutes almost one-third of all carcinoma. It usually arises from the vaginal portion of the cervix. There are two forms of this type:—the vegetative or cauliflower growth, and the nodular, or infiltrating growth. Gebhard thinks the difference due to a difference in the blood supply. When abundant the growth is superficial, and when poor, the growth is deeper and infiltrates the tissues.

Then less commonly we find the cylindrical-celled or adeno-carcinoma arising from the mucous glands of the cervix.

Both these types may be found in the cervical canal also, and here we have the most fatal form of uterine carcinoma, on account of the early infiltration of the neighboring structures.

Carcinoma of the body of the uterus is the most favorable of all for it is slow growing and invasion and metastes come late.

As to operative treatment, when the patient is seen early and the infiltration of the parametria and involvement of the surrounding structures has not gone too far, without a doubt radicalism is the word. In this direction the limit has been reached. The important principles of the operation are a wide resection of the broad ligaments, going well out to the pelvic wall on each side and carefully dissecting out the ureters, and well down on the vagina in carcinoma of the cervix so as to give the growth a good margin. The excision of the pelvic glands is a debated question, some operators favoring the step and others claiming that where the glands are involved there is such a small chance of cure that this procedure is not justified as it unnecessarily lengthens the operation. There have been some who advocated the resection of the bladders and ureters when they are involved, but this is carrying the operation too far, it seems to me.

Removal of the necrotic tissue with a curette and the use of the cautery preceding the operation is a good step in carcinoma of the cervix. This is somestimes done a week or so before the abdominal operation, but the advisability of this procedure is questioned. I saw a case where cauterization was done as a palliative measure in what was thought to be an inoperable carcinoma of the cervix, and a few days later, on vaginal examination, it was found that the induration in the parametria, which had been so marked before the cauterization, had disappeared and the uterus was freely movable and operation was comparatively simple. The induration in this case was an inflammatory thickening and not due to carcinomatous infiltration.

The history of the development of the radical operation for carcinoma of the uterus is most interesting, but I shall not go into it very fully. The operation had been attempted per vagina early as the sixteenth century, but Freund, in 1878, was the first one to do the radical abdominal operation. His operation never found favor because of the high mortality following it.

The late John Byrne of Brooklyn, in 1872, developed his vaginal operation with the galvano-cautery and it had much success in his hands. Czerny, following Freund, described a vaginal operation which for a time practically supplemented the abdominal one. But the results with it were not at all satisfactory and only a very small percentage of cures or cases that went for two years without recurrence were seen.

Then came Mackenrodt's combined vaginal and abdominal operation in 1894, and shortly after this Rumph, Ries and Clark, in 1895, each described independently a radical abdominal operation at about the same time. Wertheim and Werder, working independently, further developed this operation, giving us the operation as it stands to-day.

But statistics show that this radical operation falls far short in many cases, and the growth recurs in a large percentage. The German operators are getting the best results, because, as I said before, they get their patients early. Carcinoma of the fundus, if taken early, is very favorable and nearly 100% are cured. Of the cervix, the figures are often quite discouraging. Baldy, for example, has reported only 5% of cures. Leopold's figures are better, being 43.2%; Kaltenbach's are 13.9%, and Wertheim, I believe, gives 60% of cures in his last statistics.

We now come to the treatment of those poor unfortunates who are in the inoperable class. Where the disease has gone too far to justify a radical mutilating operation that would do no good. The best we can hope to do here is to meet the symptoms as they arise and to try to make life as comfortable as possible for the patients. There are a number of cures reported following various palliative measures, however, and from a reliable source. Czerny, Lomer and Chrobak all report such cases.

The main symptoms that we have to combat are the offensive discharge, that so often makes the patient almost unbearable to herself and to the other members of her household, the haemorrhages and the pain. The vast number of remedies and methods tried in these cases bears witness as to the inefficacy of any one of them.

I shall simply enumerate the majority of the more common methods that have been used without discussing them. Of the chemical agents injections of alcohol, turpentine, acetic acid, arsenious acid, cobra venom, etc., etc., have been put to the test. As applications under this class, various cauterants and escharotics such as carbolic acid, formalin and zinc chloride have had their advocates. These all give temporary relief, but thir action cannot be controlled and much damage may be done to the surrounding structures and then they are often quite painful. The application of powdered quinine has been given a trial.

Electricity at one time held out great hopes. Roentgen Rays were looked to as a possible source of relief for the sufferer from carcinoma. Treatment with the rays is, no doubt, very useful, and may relieve pain and retard the growth, but all reported cures from its use are questionable. A course of X-Ray treatment is advised by some as a precautionary measure following operation. "Fulguration," or the use of the high-tension spark, which promised so much at first, has failed to make good.

Cauterization with the actual cautery is probably the most general employed method. This, of course, is preceded by the removal of the necrotic tissue with the curette. The foul discharge in carcinoma of the cervix and uterus is due to the action of saprophytic bacteria upon the carcinoma cells, which do not possess the same defensive means to combat bacterial invasion as normal cells. This terrible discharge is often the most distressing feature of carcinoma, and the removal of a mass of necrotic tissue followed by cauterization always does much good. But cauterization causes necrosis and a slough occurs and in a few days there is bacterial invasion again. Thus only temporary relief is obtained.

Most of the work on carcinoma to-day is along biological and bio-chemical lines and I believe that it is from this quarter that we are to look for the solution of the problem. So far no definite results have been obtained from the point of a cure. Trypsin has failed and so have the various sera that have been used. The work of Coley, of New York, with his mixed toxins of erysipelas and bacillus prodigiosus on inoperable sarcomata is most encouraging. He has had, up to the present time, sixty-five cases of inoperable sarcomata in which the tumors disappeared under its use. At least forty-one of these remained well from three to eighteen years after the treatment was begun. This treatment has also been tried in carcinoma with variable results and in some cases seems to exert a marked inhibitory action upon the carcinoma.

Radium has been of some service and it does give symptomatic relief for a time. The pain is relieved, the discharge abated and the offensiveness diminished. I have seen it used in two cases by Dr. Howard A. Kelly. Both cases were recurrent growths and the patients improved noticeably at first, especially as regards pain.

In 1907, in the Journal of the American Medical Association for April 27th, Dr. George Gellhorn, of St. Louis, described a method of treating inoperable carcinoma of the cervix by the application of acetone. This has proved very successful and from personal experience I can recommend it.

Acetone is a transparent, colorless liquid with a penetrating, pleasant ethereal odor and a pungent, sweetish taste. It has in-tissues. It is used in the laboratory as a hardening agent and it was from this use that Dr. Gellhorn got his idea of applying it in the treatment of carcinoma.

The original technic for its use, as described by Gellhorn, is briefly as follows: For the first treatment the patient must be anaesthetized. All necrotic carcinoma is removed with the curette; the crater thus formed is packed for a short time to stop the bleeding; then it is carefully dried and, with the patient in the Trendelenberg position, one-half to one ounce of pure acetone is poured in through a tubular speculum so that it comes in direct contact with the raw crater. It is left there for from fifteen to thirty minutes. The acetone is then drained off through the speculum and the cavity is packed with narrow gauze soaked in acetone. The vulva and vagina are washed with salt solution and a dry cotton tampon inserted. The gauze is allowed to remain for several hours.

A modification of this method is to pack the crater with gauze soaked in the acetone at first after the excochleation instead of pouring in the acetone itself. This gauze may be left in for a number of hours. I have even left it in over night. It is probably better for the first treatment to apply the acetone direct as described above and use the gauze for the subsequent treatments. These subsequent treatments can be given in the office without an anaesthetic and should be given two or three times a week.

Care must be taken to avoid spilling acetone upon the skin or mucous membrane of the vulva or vagina for it causes intense burning and smarting. This may be prevented by smearing the parts with vaseline.

After the first treatment there may be some slight absorption of the acetone, as shown by the fruity odor to the breath and the appearance of acetone in the urine, but this is very rare.

The immediate effects of this treatment are that it checks the oozing, the tissue is hardened, the crater contracts down. No eschar is cast off as in cauterization. This is not a cauterization. The hardened tissue remains in situ and is probably organized like a thrombus.

The remote effects are that the terrible odor and discharge are relieved and septic absorption stopped. The haemorrhages cease and this removes a great weakening drain. The walls of the crater become smooth and firm and the tissue is no longer friable. The patient is improved in general condition, the anaemia becomes less, the appetite becomes better and there is a gain in weight.

Deep seated pain is not controlled by acetone, but in many cases there is marked improvement in this symptom and the pain decreases as the general condition improves.

There should, of course, be general hygienic treatment in connection with the local treatment, and later, as the disease advances, recourse must be had to morphia to control the pain and suffering, but this should be deferred as long as possible and other remedies tried first.

When the growth attacks the bladder and the ureters in the last stages, the patient may be made more comfortable by putting catheters in the ureters in order to avoid the discomfort of a urinary fistula. These catheters may be left in the ureters for weeks if the kidney pelvis is occasionally washed out.

I have used the acetone treatment in three cases and have seen it used in a number of others. In all of them there was marked benefit where the treatment was kept up regularly. Of course, the acetone does not give a permanent cure, but it does give noticeable relief and amelliorates the chief symptoms and makes the life of the patient more endurable. And in the majority of the cases it lengthens life by many months. Until we have discoered a cure for this disease, we shall be confronted with these inoperable cases and anything that helps us to give them such marked relief should be hailed as a great boon.

421 Candler Bldg.

